# Beyond carbon measurement: Turning agricultural carbon into credible climate action

**DOI:** 10.1016/j.xinn.2026.101531

**Published:** 2026-08-06

**Authors:** Tong Li, Xin Tian, Ram C. Dalal, Apostolos Kyriazopoulos, Amin Sharififar, Anquan Xia, Rajiv Pandey, Peng Fu, Nanthi Bolan, Jinran Wu, Geoffrey J. McLachlan, Woo-Jung Choi, Zhihong Xu, Yanfen Wang, Bojie Fu, Vilim Filipović, Scott Chapman, Yash P. Dang, Gustau Camps-Valls

**Affiliations:** 1 School of Agriculture and Food Sustainability, The University of Queensland, St Lucia, QLD 4072, Australia; 2 School of Mathematics and Physics, The University of Queensland, St Lucia, QLD 4072, Australia; 3 Laboratory of Range Science, Department of Forestry and Management of the Environment and Natural Resources, Democritus University of Thrace, Orestiada 68200, Greece; 4 Sydney Institute of Agriculture & School of Life and Environmental Sciences, The University of Sydney, Sydney, NSW 2015, Australia; 5 Development and Research Center (National Geological Archives of China), China Geological Survey, Beijing 100037, China; 6 Forest Ecology & Climate Change Division, ICFRE-IFP, Ranchi, Jharkhand 835 303, India; 7 Division of Forestry Statistics, ICFRE, Dehradun, Uttarakhand 248 006, India; 8 School of Plant, Environmental and Soil Sciences, Louisiana State University AgCenter, Baton Rouge, LA 70803, USA; 9 UWA School of Agriculture and Environment, The University of Western Australia, Perth, WA 6009, Australia; 10 Department of Rural & Biosystems Engineering (Brain Korea 21), Chonnam National University, Gwangju 61186, Republic of Korea; 11 AgriBio Institute of Climate Change Management, Chonnam National University, Gwangju 61186, Republic of Korea; 12 Centre for Planetary Health and Food Security, School of Environment and Science, Griffith University, Nathan, QLD 4111, Australia; 13 College of Resources and Environment, Beijing Yanshan Earth Critical Zone National Research Station, University of Chinese Academy of Sciences, Beijing 100049, China; 14 State Key Laboratory of Regional and Urban Ecology, Research Center for Eco-Environmental Sciences, Chinese Academy of Sciences, Beijing 100085, China; 15 Image Processing Lab (IPL), Universitat de València, 46980 Valencia, Spain

Climate mitigation debates increasingly extend beyond technical feasibility to questions of public understanding, institutional credibility, and practical implementation. Recent work in *Science* emphasizes translating mitigation concepts into actions society can understand, evaluate, and adopt.^1^ This focus aligns with the Paris Agreement, the 2030 Agenda for Sustainable Development, and calls to place soil security and climate-smart agriculture at the center of climate, food, and water agendas.^2^^,^^3^ Agriculture is therefore central to credible climate action.

Agriculture contributes substantially to greenhouse gas (GHG) emissions and offers important opportunities for carbon sequestration, land stewardship, and broader sustainability transitions.^2^^,^^3^ Advances in carbon measurement and accounting, together with improved monitoring, reporting, and verification (MRV), are making agricultural emissions and removals more transparent. Yet measurability alone does not produce credible carbon credits or durable climate action. Evidence must be interpreted through methodological rules, institutional arrangements, and implementation conditions that account for ecological variability, uncertainty, and farm-level realities.^2^^,^^3^ This creates a governance gap: measurement capabilities are advancing faster than institutions can translate measurable carbon into credible claims and durable action. This commentary therefore argues for an integrity-centered governance framework. Measurement-centered approaches ask whether agricultural carbon outcomes can be observed, quantified, reported, and verified. Integrity-centered governance asks how those measurements can support credible claims and durable action through methodological rules, uncertainty treatment, verification procedures, risk allocation, and sustained farmer participation. Figure 1 summarizes this transition.

**Figure 1:**
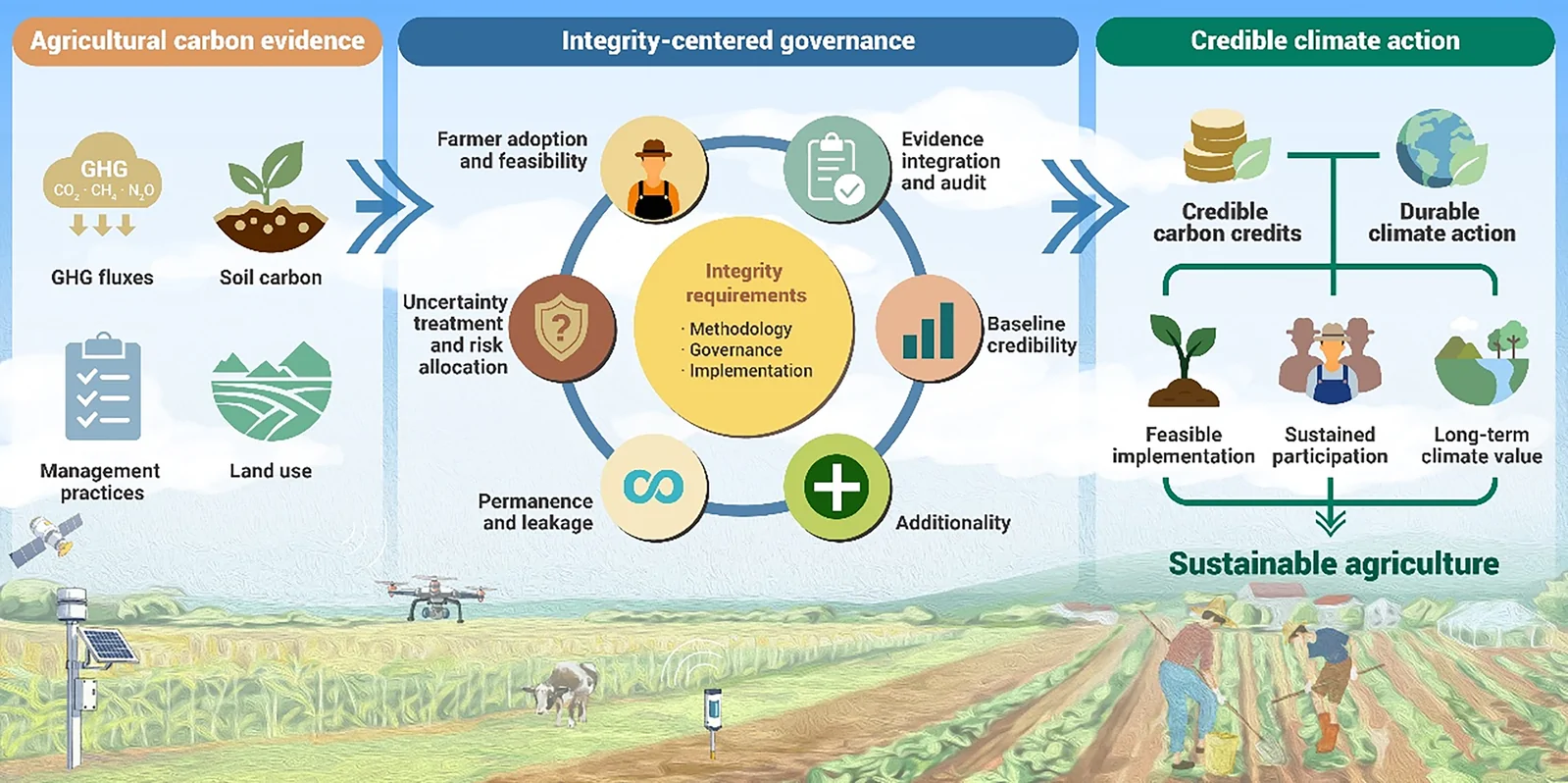
Framework linking agricultural carbon evidence and integrity-centered governance to credible carbon credits, durable climate action, and sustainable agriculture

## Measurement has outpaced governance

Agricultural carbon measurement now integrates ground instruments, drones, aircraft, and satellites to monitor management, vegetation, soil and crop conditions, and emissions-related signals. Proximal sensing, including near- and mid-infrared, X-ray fluorescence, and gamma-ray spectroscopy, enables rapid, high-resolution soil carbon assessment. These approaches strengthen the evidence base and inform additionality, permanence, and leakage, but crediting still depends on governance rules.

Institutional arrangements have not kept pace. Registries and project methodologies often do not fully integrate evidence from remote sensing, soil sampling, flux measurements, statistics, and process-based models. The challenge is how these streams are weighted, audited, and translated into crediting decisions, including uncertainty treatment, baselines, additionality, permanence, leakage, and risk allocation. This institutional translation gap constrains credible climate action.

## Agriculture is not a simple carbon sector

The measurement-governance gap is especially pronounced in agriculture. Agricultural emissions and removals are harder to quantify and standardize than emissions from many energy and transport activities because they arise from interactions among soil, livestock, vegetation, climate, management, and time. These biologically mediated dynamics are sensitive to local conditions.^2^^,^^3^ Practices such as cover crops, reduced tillage, and livestock interventions can therefore yield different net climate effects across contexts, reflecting factors such as methane and nitrous oxide trade-offs, soil drainage, feed composition, and climate. The key question is whether a measured change represents a genuine and durable net climate benefit attributable to the intervention.

This variability poses a structural challenge for markets seeking standardized, interchangeable credits. Agricultural carbon outcomes are context dependent, time varying, and often slow to manifest.^2^^,^^3^ Spatial heterogeneity complicates baselines and comparability; GHG trade-offs complicate net mitigation claims; and delayed or reversible soil carbon responses complicate permanence. Observed changes may also reflect weather, markets, policy incentives, or pre-existing management trends. MRV can document change, but governance rules determine whether that change should be credited.

## Measurable carbon does not guarantee credible credits

Measuring emissions and removals at scale does not automatically make agricultural carbon credits credible or legitimate. Beyond measurement accuracy, credit integrity depends on baseline credibility, additionality, permanence, leakage, and uncertainty treatment. These conditions concern how the no-project scenario is defined, whether the change would have occurred without carbon incentives, whether benefits endure, whether emissions are displaced elsewhere, and how measurement, model, baseline, and reversal uncertainties affect credit issuance and risk allocation. Crediting therefore depends on methodological and institutional rules that determine which claims are supported by data and modeling outputs.^2^^,^^3^

Remote sensing and other MRV tools can inform these judgments by providing evidence on land use, practice implementation, spatial heterogeneity, and environmental conditions. Such evidence can strengthen crediting systems but cannot establish causality, permanence, or integrity on its own. Crediting systems may also favor readily quantified carbon outcomes while overlooking soil function, resilience, biodiversity, and livelihood stability, even though these factors shape whether mitigation endures.^2^^,^^3^ Measurability is necessary for credibility but insufficient to ensure that credited interventions are legitimate, robust, or sustainable.

## Farmer adoption as a condition of climate integrity

Agricultural climate mitigation depends on practices implemented and sustained by farmers, land managers, and other actors across working landscapes, not on models or registries alone. Adoption is therefore a condition of climate integrity, not merely a matter of promotion or outreach. Even scientifically sound interventions may be difficult to adopt or sustain when implementation is complex, monitoring costs are high, returns are uncertain, or program requirements conflict with agronomic priorities and farm-level realities. Soil carbon sequestration should be framed as “carbon for soil” rather than “soil for carbon.” Under this framing, carbon incentives should support soil health, productivity, resilience, and long-term land stewardship; soil should not be reduced to a credit-generating asset.^2^^,^^3^

Adoption affects credit integrity in three ways. First, additionality depends on whether programs induce management changes beyond what farmers would otherwise have done. Second, permanence depends partly on incentives, technical support, and contracts that sustain practices; otherwise, gains may be reversed or mitigation may not persist. Third, legitimacy weakens when monitoring burdens and the costs associated with uncertainty and reversal risk fall disproportionately on farmers. Credits may meet methodological criteria yet remain socially fragile and have limited climate value when programs fail to provide viable incentives, enable feasible implementation, or sustain participation. Evaluation should therefore consider the amount of carbon credited alongside the durability of participation under actual farming conditions.

## From better MRV to better climate governance

The next challenge is to match advances in measurement with governance arrangements capable of using evidence credibly. In agricultural carbon systems, uncertainty cannot remain a residual technical concern; clear rules must specify how it affects evidence thresholds, credit issuance, verification standards, and risk allocation. Credibility therefore requires robust quantification and transparent procedures for using imperfect but policy-relevant evidence. Remote sensing, soil measurements, and model-based approaches are useful for identifying management practices, monitoring environmental conditions, and supporting independent verification.^4^ Their value for crediting depends on whether institutions incorporate this evidence into project methodologies, audit procedures, and crediting rules.

The Australian Carbon Credit Unit (ACCU) Scheme provides a concrete example of this institutional translation. A comparison of major standards for Australian soil carbon projects indicates that credit quality depends on quantification and on how protocols address additionality, permanence, baselines, safeguards, assessment, verification, and crediting periods.^5^ Measurement and modeling can support credit issuance only when approved methods specify how evidence is evaluated, verified, and translated into creditable abatement. The critical transition is therefore not from low precision to high precision but from scientific capability to operational legitimacy.

Governance must also align incentives and evaluation criteria with the broader conditions under which agricultural mitigation endures. Carbon program evaluation should consider credit volume alongside implementation feasibility, sustained participation, and long-term climate value. In agriculture, relevant forms of value include resilience, soil function, water retention, nutrient cycling, and adaptive capacity.^2^^,^^3^ Governance frameworks focused narrowly on carbon metrics risk overlooking the conditions that make mitigation durable and socially legitimate. MRV therefore matters as a governance tool, not an endpoint: its climate value depends on systems that connect measurement with sustained adoption and credible action.

## Conclusion

Agricultural carbon credits can mobilize investment, improve land management, and contribute to climate mitigation, but credibility depends on more than measurement precision. In agriculture, ecological variability, methodological uncertainty, institutional interpretation, and sustained farmer adoption shape whether carbon claims are credible. Measurable carbon becomes credible climate action only when governance manages uncertainty, defines transparent crediting rules, allocates risk, aligns incentives with farm-level realities, and recognizes soil function, resilience, and livelihood stability alongside carbon outcomes. Measurement is meaningful when embedded in institutions that support legitimate crediting, feasible implementation, sustained participation, long-term climate value, and sustainable agriculture.

## Funding and acknowledgments

This research was funded by the 10.13039/100032049National Foundation for Australia-China Relations, grant number NFACR240313. The funders had no role in the study design, data collection and analysis, decision to publish, or preparation of the manuscript.

## Declaration of interests

The authors declare no competing interests.
